# Gross Motor Coordination: We Have a Problem! A Study With the Körperkoordinations Test für Kinder in Youth (6–13 Years)

**DOI:** 10.3389/fped.2021.785990

**Published:** 2021-12-10

**Authors:** Matteo Giuriato, Valentina Biino, Marianna Bellafiore, Giuseppe Battaglia, Antonio Palma, Carlo Baldari, Laura Guidetti, Maria Chiara Gallotta, Federico Schena, Massimo Lanza

**Affiliations:** ^1^Department of Human Sciences, University of Verona, Verona, Italy; ^2^Unit of Molecular Biology, Department of Health and Natural Sciences, Faculty of Physical Culture, University of Physical Education and Sport, Gdansk, Poland; ^3^School University of Medicine and Surgery, University of Verona, Verona, Italy; ^4^Sport and Exercise Sciences Research Unit, University of Palermo, Palermo, Italy; ^5^Department of Theoretical and Applied Sciences, eCampus University, Novedrate, Italy; ^6^Department of Unicusano, University Niccolò Cusano, Rome, Italy; ^7^Department of Physiology and Pharmacology “Vittorio Erspamer”, Sapienza University of Rome, Rome, Italy; ^8^Department of Neurosciences, Biomedicine and Movement Sciences, University of Verona, Verona, Italy

**Keywords:** gross motor coordination, KTK, youth development, children, physical activity, health, motor coordination (MC)

## Abstract

The main goal of our cross-sectional research was to determine the current values of gross motor coordination (GMC) of Italian boys and girls between 6 and 13 years of age. Secondary goals were to study gender differences, and the four subtests trend with ages. Results were compared with the references proposed by KTK authors and with similar searches. Anthropometric measurements and KTK data from 2,206 schoolchildren (girls: *n* = 1,050; boys: *n* = 1,156) were collected. The KTK raw score (RS) increased with the age of the subjects (*r* = 0.678; *p* < 0.001). In 11–13-year-old subjects, the increase in results is less than in younger subjects. RS showed differences by gender (*F* = 5.899; *p* = 0.015) and age (*F* = 269.193; *p* < 0.001) without interaction gender × age. Motor quotient (MQ) tended to decrease with age (*r* = −0.148; *p* < 0.001); it showed differences by gender (*F* = 79.228; *p* < 0.001), age (*F* = 14.217; *p* < 0.001), and an interaction gender × age (*F* = 2.249; *p* < 0.05). Boys showed better performance than did girls in the raw scores of three of four subtests (JS: *F* = 24.529; MS: *F* = 9.052; HH: *F* = 11.105). Girls show better performances than did boys in the WB (*F* = 14.52). Differences between genders make us believe it appropriate to maintain a differentiated standardization. RS increased with age, and it seems reasonable, therefore, to maintain a GMC age-based normalization. On the contrary, MQ tended to decrease. All this makes us speculate that today's young people accumulate less significant motor experiences over the years compared to those achieved by their peers in the 1970s. Italian data were lower than German references and Belgian results but slightly higher than the Brazilian ones. The comparison among these four searches confirmed a worrying downward trend in GMC and its characterization by geographical and sociocultural areas. Updated parameters of the KTK can provide helpful references to improve policies to support physical activity, sport, and physical education in youth.

## Introduction

Motor learning and control characterize children development and their adaptation to the physical and social environment. The manifestation of motor learning and control is the children's motor competence. It can be defined as mastery in fundamental movement skills (e.g., walking, throwing, and catching) and in more specialized movement sequences such as lifelong physical activity abilities like cycling, swimming, or sport-specific skills (1). Motor abilities are articulated in basic stability (static or dynamic balance), manipulation, object control, and locomotor abilities (2). Basic stability and locomotor abilities, often defined as “gross motor coordination” (GMC), involve the control of two or more body segments and/or the global movement of the body in space (3). These two aspects of movement are fundamental both in the acquisition of fundamental motor skills (FMS) and in the development of specialized movements and techniques of daily life and sport. FMS is generally categorized into basic locomotor skills that lead children to transfer the body in space (e.g., walking and running) and object control skills that allow them to manipulate and project objects (i.e., striking, kicking, etc.) (4). In both cases, both the stability and the coordination of the body segments are necessary for a mastery of the movements (2).

The GMC is essential for acquiring both advanced control of FMS and that of specialized movement techniques. Consequently, it is also necessary for the training of health-related physical abilities, such as strength, endurance, and flexibility, and for those related to sports performance. GMC provide the basis to reaching a high level of motor competence (MC), to develop adequately, maintain health, and gain athletic excellence (5). GMC, therefore, plays a crucial role in the development and active lifestyle (6). Children with a high level of coordination are more involved in physical activity (PA) and sport and tend to reach better performance (7, 8). On the other hand, children with a low level of coordination are less inclined to participate in physical activities (9–11). Numerous studies confirm the relationship between low motor skill levels in children, poor PA, and increased BMI during developmental age (12–14), especially in girls (15). Physical inactivity leads to a worsening of body weight and fat mass with a negative influence on GMC tasks (15, 16). Data from Lopes et al. (17) showed that clumsy children have higher BMI levels more frequently. The term GMC, in this paper, will be used to refer to the ability to execute a wide range of motor activities involving whole-body movement (3, 18).

GMC in childhood influences, directly and indirectly, health-related physical fitness and the development of long-term health outcomes in children and adolescents (19). Several researchers (11, 20–23) studied the coordination role in promoting health, yet it is still an open question. Poor GMC prevents children from reaching a good level of motor skills (24) and, consequently, does not allow them to participate safely and vigorously in sports practices (10). On the contrary, mastering motor skills in childhood seems to help children participate regularly in organized sports and spontaneous physical activities (12, 15, 25–28).

In the vast domain of exercise for health, the relationship between motor coordination and cognitive development also begins to show significant evidence (29, 30). Findings support the association between motor coordination and executive function in childhood (31, 32), adolescence (33) and in all other phases of life (34). Marchetti et al. (35) suggests that the cognitive demands of complex movement and sport tasks, as well as sensorimotor learning, may be responsible for the positive association of PA and sports with higher-level cognition and metacognition.

Despite this mass of evidence that assigns coordination a significant role in movement education and health promotion, there are some serious obstacles to study and structurally promote this aspect of motor skills. Assessing GMC is complex because it manifests itself in countless modes of movement: locomotion, object control, postural stability, and dynamic balance. Stability represents the most basic of the movement and sport. For this reason, stability begins to develop early in life and often children who are exposed to a variety of movement experiences have no difficulty developing fundamental stability abilities (2). Several literature reviews (1, 36–39) identify a second obstacle in the coordination study: the lack of a reference test shared by the entire scientific community. Several tests' batteries have been proposed and validated, some with the specific purpose of identifying subjects with coordination difficulties, others for measuring different aspects of coordination, others still applicable to FMS. Among these batteries, we find significant differences related to the type of movements investigated and the age groups in which they can be applied. Only some batteries provide standard values for girls and boys (36). All this results in the lack of a “Gold standard” to assess coordination. It is relevant to overcome these difficulties also from an educational perspective to promote motor literacy in young people. Indeed, PA guidelines for youth (40–44) indicate the necessity to develop a large patrimony of motor skills and a positive attitude toward motor learning. These goals, however, cannot be properly pursued and documented in the absence of a shared way to measure them. Lastly, since the assessment of GMC at a young age must be applied to a large number of people, such as in physical education classes and sports training, the assessment tools need the characteristics of applicability and simplicity of execution (36). Vandorpe et al. (45) suggested that GMC cannot be assessed independently from the pure fitness characteristics (e.g., strength, speed, endurance, and flexibility). Further, in terms of health, the physical components in relation to GMC have been evaluated extensively (45). The Körperkoordinations Test für Kinder (Body Coordination Test for Children) (46, 47) seems to be one of the batteries that can meet the requirements described above (48). It was conceived and validated, with a population of 1,228 German subjects between 5 and 14 years of age by Kiphard and Schilling in 1974 (46) and verified in 2007. The four subtests make it up to measure different aspects of the GMC, not related to specific sports skills (3). Gross motor coordination is always measured in association with some of the physical abilities such as strength, speed, endurance, and flexibility (45). Most test batteries consist of elements that measure both physical and coordination skills (49). KTK also measures GMC in movements that also involve strength and speed characteristics (46, 47). The four subtests are “Walking backwards” (WB), “Jumping sideways” (JS), “Moving sideways” (MS), and “Hopping for height” (HH). The four subtests are used, with the same parameters, in all ages of test application, allowing, and so also longitudinal studies. The test takes about 20 min per child. The KTK is a battery suitable in different fields like physical education, sports, health promotion (48), and talent identification (50).

Some limitations of the KTK are the lack of indications for the control of objects, the tendency to overestimate the number of children with GMC problems, and the comparison with standardized values based on data from 1974 that may be obsolete (48). This last point can, however, be considered also a positive element because it allows the study of GMC's change over long periods.

GMC plays a critical role in youth development and active lifestyle, but these parameters are, however, lacking, particularly in Italy where, to our knowledge, only surveys have been carried out on small groups (51–55) or in pre-school age (56, 57).

The main goal of our cross-sectional research was to verify for the first time the current values of GMC of Italian boys and girls between 6 and 13 years of age based on a large cohort of Italian children. Our results were compared with the values proposed by Kiphard and Schilling (46) and with similar researches that used the KTK in more recent years (45, 58). Based on our previous unpublished studies as well as Vandorpe et al. (45) and Moreira et al. (58) data, it was assumed that the current coordination levels of Italian boys and girls are lower than the reference values of the KTK battery. Secondary goals were to study gender differences, and the four subtests trend with ages. In all four subtests, we assume an increase in raw values that slowed down with increasing age. We expected that girls exhibit similar or higher values than boys in the WB. In the JS and HH, boys should show overall values higher than those of girls due to higher strength levels. There are no known reasons to assume gender difference in MS.

## Methods

### Participants

Two thousand two hundred six schoolchildren (girls: *n* = 1,050; boys: *n* = 1,156), aged between 6 and 13 years (Table 1), from 49 primary and lower secondary schools (private and public), representative of the Italian geographical areas (North Italy = Veneto; Center Italy = Lazio; South Italy = Sicily) were randomly recruited in this cross-sectional study (convenient sample), including urban and rural areas. The included schools were equipped with appropriate and similar sports facilities to conduct comparable measurements. Sample procedures considered the total number of schools (private and public), geographic regions (north, center, south), and urban and rural places. The population examined is similar to or greater than that of similar studies (45–51). The measurements were conducted from January 2019 to February 2020. The Ethical Boards of the Universities of Verona (N. 2019-UNVRCLE-0298910) and Palermo (N. 8/2019) as well as the Institutional Review Board of the University of Rome “Foro Italico” approved the study. The study complies with the criteria for the use of people in research defined in the Declaration of Helsinki. Moreover, school principals provided further research authorizations. After researchers explained the purpose of the investigation and the research methodology, all parents provided written informed consent before participating in the study. All the measures were taken during the Physical Education lessons as scheduled in the morning framework (8.00–12.00 a.m.). All assessments were carried out by trained supervisors (Physical Education teachers or specifically trained Sport Science's students) in the same gym school context. The presence and collaboration of the curricular PE teachers were guaranteed at any time to meet the confidence of the students. The trainer–pupil ratio was 1:10.

**Table 1:** Number of subjects, mean values of raw score (RS), and motor quotient (MQ) by gender and age.

		**All sample**	**Girls**	**Boys**	***P* for gender**
6 years	Number	253	123	130	
	RS	108.97 (30.84)^∧^	105.71 (30.50)	112.05 (30.95)	n.s.
	MQ	88.42 (14.99)	83.07 (14.60)^*^	93.48 (13.57)^∧^	<0.001
7 years	Number	285	150	136	
	RS	135.96 (36.19)^∧^	133.23 (35.97)	138.97 (36.34)	n.s.
	MQ	91.76 (16.11)	88.60 (16.27)^∧^^°^^#^	95.24 (15.24)^*^^∧^	<0.001
8 years	Number	366	184	182	
	RS	151.89 (38.26)^∧^	150.18 (36.44)	153.82 (40.05)	n.s.
	MQ	88.50 (16.29)	87.32 (15.48)^∧^^§^^%^	89.69 (17.03)^#^	n.s.
9 years	Number	357	163	194	
	RS	175.36 (41.88)^∧^	173.07 (41.76)	177.28 (42.00)	n.s.
	MQ	87.06 (17.01)	82.79 (16.88)^*^	90.65 (16.32)^°^	<0.001
10 years	Number	370	178	192	
	RS	188.51 (39.07)^∧^	189.46 (38.46)	187.64 (39.70)	n.s.
	MQ	84.30 (15.44)	82.30 (15.60)^*^	86.16 (15.09)	<0.05
11 years	Number	223	99	124	
	RS	205.23 (33.34)^∧^^&^	203.40 (31.72)	206.69 (34.63)	n.s.
	MQ	86.89 (14.45)	84.78 (12.98)^∧^	88.57 (15.37)	n.s.
12 years	Number	218	99	119	
	RS	211.76 (31.61)^∧^^&^	210.85 (30.18)	212.51 (32.85)	n.s.
	MQ	81.25 (15.58)	78.25 (15.08)	83.75 (15.60)	<0.05
13 years	Number	133	54	79	
	RS	221.53 (32.01)^∧^^&^	216.02 (32.32)	225.30 (31.44)	n.s.
	MQ	80.89 (17.47)	75.02 (16.78)	84.90 (16.88)	<0.001
All years	Number	2,206	1,050	1,156	
	RS	170.35 (49.83)	166.89 (49.49)	173.49 (49.95)	<0.05
	MQ	86.64 (16.22)	83.72 (15.92)	89.29 (16.04)	<0.05
Age	RS		*F* = 269.193; *p* < 0.001		
	MQ		*F* = 14.217; *p* < 0.001		
Gender	RS		*F* = 5.899; *p* = 0.015		
	MQ		*F* = 79.228; *p* < 0.001		
Ages × gender	RS		*F* = 0.488; *p* = n.s.		
	MQ		*F* = 2.249; *p* = 0.028		

*All sample:*

∧ *p < 0.001 between all couples*,

& *p = n.s. 13 vs. 12 and 12 vs. 11*.

Girls: *p <0.01 vs. 13 years,

∧ *p < 0.001 vs. 13 years*,

° *p < 0.01 vs. 9 years*,

# *p <0.01 vs. 10 and 12 years*,

§ *p < 0.01 vs. 10 years*,

% *p < 0.001 vs. 12*.

Boys: *p <0.01 vs. 8 and 11 years,

*^∧^p < 0.001 vs. 10, 12, and 13 years, °p <0.001 vs. 12 years*,

*^#^p < 0.01 vs. 12 years*.

### Anthropometric Measurements

To ensure that the subjects correctly represented the Italian population with regard to the proportion of underweight, normal weight, overweight, and obese subjects, weight and height measurements were collected. Anthropometric measurements were taken according to the standard procedures described by the International Society for the Advancement of Kinanthropometry (59). Height was measured with a stadiometer to the nearest 0.5 cm. Weight was measured to the nearest 0.1 kg with an electronic scale with the subject wearing minimal clothing. Children were classified as underweight, normal weight, overweight, and obese using age- and gender-specific cutoff points (60, 61).

### Gross Motor Coordination Measurements

GMC was evaluated through Körperkoordinations Test für Kinder, referred to as KTK (46, 47), which consisted of four items:

1. Walking backwards—WB: walking backwards three times along each of three balance beams (3 m length; 6, 4.5, and 3 cm width, respectively; 5 cm height). A maximum of 24 steps (eight per trial) was counted for each balance beam, which comprises a maximum of 72 steps (24 steps × 3 beams) for this test.
2. Jumping sideways—JS: jumping laterally as many times as possible over a wooden slat (60, 4, and 2 cm) in 15 s. The number of jumps over two trials was summed.
3. Moving sideways—MS: moving across the floor in 20 s by stepping from one plate (25 × 25 × 5.7 cm) to the next, transferring the first plate, stepping on it, etc. The number of relocations was counted and summed over two trials.
4. Hopping for height—HH: jumping from one leg over an increasing pile of pillows (60 × 20 × 5 cm each) after a short run-up. Three, two, or one point(s) were/was awarded for successful performance on the first, second, or third trial, respectively. A maximum of 39 points (ground level plus 12 pillows) could be scored for each leg, yielding a possible maximum score of 78.

KTK test and its scoring were carried out according to the authors' indications (46, 47). Children were tested alone or in small groups, and the tasks were performed one child at a time, during the physical education lessons, in the school gymnasium. Before each test, the children received an oral explanation about the procedure. For the raw score on the total test battery, a test–retest reliability coefficient of 0.97 was reported. For the four subtests, based on the raw score, sufficiently reliable coefficients were reported as well (WB: 0.80; MS: 0.84; HH: 0.96; JS: 0.95). Intercorrelations between the four subtests varied from 0.60 (WB/JS) to 0.81 (HH/JS) for the reference group of 1,228 children. Factor analysis revealed that the four subtests all load on the same factor, namely, GMC. The percentage of total variance of the KTK explained by the four subtests varied from 80.9 (age 6) to 97.7 (age 9) (46, 47). The KTK allows an objective and straightforward evaluation of a child's gross motor coordination only, with only limited interference of the child's physical fitness, which discriminates this test from most other instruments. The raw test scores from each of the four subtests can be transformed into gender- and age-specific motor quotients value (MQ), which were based on the performance of 1,228 normally developing German children in 1974. Scoring of the KTK test was performed according to the manual (47). The mean standardized value is 100 with a standard deviation of 15 (46, 47). MQ describes the level of GMC (45, 47), and values between 85 and 115 describe the normality (**Table 6**).

The measurements obtained from the KTK are the following:

- The raw results of the four tests that make up the KTK (WB raw, MS raw, JS raw, and HH raw);
- The sum of the four subtests raw values called “Raw score” (RS);
- The four normalized values of the subtests (WB, MS, JS, and HH), obtained from the normalization tables for age and gender (41, 42). The mean of each of the four standardized values is 100 with a standard deviation of 15;
- The sum of the four standardized values (MQ raw);
- The “Motor Quotient” (MQ), obtained from the standardization tables. It summarizes the overall normalized value of gross motor coordination measured with the KTK.

In our research, we considered all the above measures except the sum of the standardized values of the four tests, which are largely represented by MQ.

### Statistical Analysis

The Shapiro–Wilk-test for normality was initially used to evaluate the distribution of data. Number frequency was displayed to describe age and gender classes. The chi-square test was carried out to study any significance between frequencies of gender according to age groups. The scores of the KTK were calculated and showed as means and SDs by age and gender. A Spearman correlation was used for not normally distributed data and Pearson correlation for normally distributed data. The two-way ANOVA was run to examine if there was an interaction effect between the independent variables age and gender on all KTK scores. The comparison between the results of the various researches was carried out with one-way ANOVA using the values of the means, the number of subjects, and the standard deviations. The results of ANOVA were displayed through the F-values; significant interaction and main effects were examined with Bonferroni *post-hoc t*ests. Level of significance was set at *p* < 0.05. The software SigmaStat for Window, version 3.5 (Systat Software Inc., Erkrath, Germany), was used to perform the statistical analyses.

## Results

### RS and MQ Analyses

Table 1 illustrates the characteristics of the subjects involved in the study that presents a homogeneous distribution by age and gender. It was also verified that the prevalence of underweight, normal-weight, overweight, and obese children was consistent with recent data on Italian children of the same age (62). In particular, the four BMI categories presented the following percentages: underweight = 8%, normal weight = 61%, overweight = 22%, obese = 9%. Table 2 describes the mean values of all KTK parameters and their correlations with the age of the subjects.

**Table 2:** Means and standard deviation of all parameters of the KTK (RS, MQ, WB raw, WB, MS raw, MS, JS raw, JS, HH raw, and HH) and their “Spearman rank order correlation” with age.

		**MQ**	**RS**	**WB raw**	**WB**	**JS raw**	**JS**	**MS raw**	**MS**	**HH raw**	**HH**
	**Means (sd)**	86.64	170.35	38.64	88.33	53.87	99.28	34.02	80.68	43.81	90.76
		(16.22)	(49.83)	(16.92)	(17.27)	(17.40)	(18.27)	(8.85)	(18.90)	(20.83)	(20.89)
Age	Correlation coefficient (*r*)	−0.148	0.678	0.459	0.078	0.674	0.006	0.298	−0.331	0.545	−0.103
	*p*	<0.001	<0.001	<0.001	<0.001	<0.001	n.s	<0.001	<0.001	<0.001	<0.001
		Number of samples = 2,206									

RS increased with the age of the subjects (*r* = 0.678; *p* < 0.001) progressively reducing, however, the increase. Up to 11 years, the annual differences are always significant, while subsequently, it is between 11 and 13 years. RS shows differences by gender (*F* = 5.899; *p* = 0.015) and age (*F* = 269.193; *p* < 0.001) without interaction gender × age (*F* = 0.48; *p* = n.s.). Overall, boys show higher RS values than girls (girls = 166.89 ± 49.49; boys = 173.49 ± 49.95; *F* = 5.899; *p* < 0.05), but the gender comparison for each age group does not show significant differences in any of the eight pairs (Table 1).

MQ tends to decrease with age (*r* = −0.148; *p* < 0.001) and shows differences by gender (*F* = 79.228; *p* < 0.001), age (*F* = 14.217; *p* < 0.001), and an interaction gender × age (*F* = 2.249; *p* < 0.05). Among the girls, 13-year-olds show a lower mean (*p* < 0.01 ÷ 0.001) than all the others except for the 12-year-olds; 7-year-old girls have better values than 9–13-year-olds (*p* < 0.01 ÷ 0.001); and 8-years-olds have better values than 10-year-olds (*p* < 0.01). Seven-year-old boys have better values than 8-, 10–13-year-olds (*p* < 0.01 ÷ 0.001); 6-year-olds have better values than 10-, 12-, 13-year-olds (*p* < 0.001); and 12-year-olds show a lower mean (*p* < 0.01 ÷ 0.001) 6–9-year-olds. In the analysis by age groups, boys always show higher values than girls except at 8 and 11 years.

### Analysis of the Four Subtests: WB, MS, JS, and HH

Tables 3, 4 show, respectively, the raw score and standardized values of the four KTK subtests for gender and age. In the raw values of the four subtests, significant differences (*p* < 0.001 ÷ 0.01) by gender and age emerged without interaction gender × age. In standardized values of all subtests, significant differences (*p* < 0.001) by gender and age were found, with interactions gender × age in JS (*p* < 01) and HH (*p* < 0.001).

**Table 3:** Raw score (mean ± standard deviation) of the four KTK subtests (walking backwards, moving sideways, jumping sideways, and hopping for height) for gender and age.

	**6**	**7**	**8**	**9**	**10**	**11**	**12**	**13**	**Age**	**Gender**	**Age × gender**
**Walking backwards raw score**											
Girls	26.84 (12.95)	30.35 (13.46)	36.04 (15.78)	41.28 (16.46)	44.15 (15.77)	48.63 (14.74)	52.72 (15.04)	48.67 (15.71)	*F* = 89.465 *p* < 0.001	*F* = 14.52 *p* < 0.001	*F* = 0.519 *p* = n.s.
Boys	25.65 (12.76)	28.20 (15.88)	31.99 (15.15)	37.79 (14.37)	40.69 (15.44)	45.53 (14.05)	48.99 (15.24)	49.29 (14.67)			
Total	26.23 (12.84)	29.33 (14.67)	34.03 (15.58)	39.39 (15.43)	42.35 (15.67)	46.91 (14.41)	50.68 (15.23)	49.04 (15.05)			
**Jumping sideways raw score**											
Girls	31.54 (10.22)	40.96 (12.94)	46.56 (13.27)	52.77 (13.03)	58.17 (12.27)	63.22 (12.13)	67.63 (11.37)	71.87 (12.61)	*F* = 266.349 *p* < 0.001	*F* = 24.529 *p* < 0.001	*F* = 0.487 *p* = n.s.
Boys	34.09 (11.70)	42.90 (11.70)	50.22 (12.60)	56.31 (13.50)	59.86 (13.66)	66.40 (13.38)	72.16 (14.06)	73.65 (13.56)			
Total	32.85 (11.05)	41.88 (12.39)	43.38 (13.06)	54.69 (13.38)	59.05 (13.02)	64.99 (12.91)	70.10 (13.08)	72.92 (13.16)			
**Moving sideways raw score**											
Girls	25.70 (5.89)	31.07 (7.17)	33.71 (6.97)	34.14 (7.46)	35.91 (7.47)	34.49 (7.45)	36.46 (10.05)	37.17 (10.10)	*F* = 50.91 *p* < 0.001	*F* = 9.052 *p* < 0.01	*F* = 0.97 *p* = n.s.
Boys	26.64 (7.30)	31.46 (7.64)	35.16 (7.77)	36.35 (8.24)	36.35 (7.76)	36.08 (9.22)	35.77 (10.64)	39.80 (12.72)			
Total	26.18 (6.65)	31.26 (7.39)	34.43 (7.40)	35.34 (7.96)	36.14 (7.61)	35.38 (8.50)	36.09 (10.36)	38.73 (11.76)			
**Hopping for height raw score**											
Girls	21.63 (11.04)	30.85 (15.30)	33.88 (16.39)	44.87 (20.29)	51.23 (19.48)	57.06 (17.50)	54.04 (15.49)	58.31 (15.46)	*F* = 136.186 *p* < 0.001	*F* = 11.105 *p* < 0.001	*F* = 0.903 *p* = n.s.
Boys	25.68 (11.70)	36.40 (14.68)	36.23 (18.84)	46.83 (21.20)	50.73 (20.57)	58.68 (17.64)	55.59 (15.48)	62.57 (14.60)			
Total	23.71 (11.54)	33.49 (15.24)	35.05 (17.67)	45.94 (20.79)	50.97 (20.03)	57.96 (17.56)	54.89 (15.47)	60.84 (15.04)			

**Table 4:** Standardized values (mean ± standard deviation) of the four KTK subtests (walking backwards, moving sideways, jumping sideways, and hopping for height) for gender and age.

	**6**	**7**	**8**	**9**	**10**	**11**	**12**	**13**	**Age**	**Gender**	**Age × gender**
**Walking backwards**											
**(WB)**											
Girls	91.10 (14.88)	87.93 (14.87)	87.33 (17.21)	88.99 (17.47)	89.95 (16.94)	93.23 (17.19)	96.10 (19.94)	87.70 (21.31)	*F* = 6.683 *p* < 0.001	*F* = 14.695 *p* < 0.001	*F* = 0.592 *p* = n.s.
Boys	89.68 (14.64)	85.50 (17.55)	82.94 (16.54)	85.41 (15.32)	86.25 (16.45)	89.51 (16.54)	90.66 (20.84)	88.63 (19.87)			
Total	90.37 (14.74)	86.78 (16.22)	85.15 (17.00)	87.05 (16.41)	87.98 (16.76)	91.16 (16.90)	93.13 (20.57)	88.26 (20.39)			
**Jumping sideways**											
**(JS)**											
Girls	90.92 (15.85)	96.66 (18.80)	98.02 (17.02)	88.60 (18.85)	89.85 (16.70)	95.12 (15.63)	92.55 (17.62)	90.54 (15.16)	*F* = 6.638 *p* < 0.001	*F* = 257.363 *p* < 0.001	*F* = 2.944 *p* < 0.01
Boys	102.00 (16.09)	108.26 (15.61)	105.76 (16.31)	106.80 (16.05)	101.15 (17.62)	104.94 (17.28)	106.01 (18.72)	106.28 (18.14)			
Total	96.61 (16.88)	102.18 (18.27)	101.87 (17.09)	98.49 (19.59)	95.71 (18.07)	100.58 (17.24)	99.89 (19.39)	99.89 (18.62)			
**Moving sideways**											
**(MS)**											
Girls	84.27 (13.58)	88.76 (16.67)	85.80 (16.54)	79.93 (15.42)	76.63 (13.85)	71.66 (15.73)	68.60 (20.42)	67.48 (21.69)	*F* = 50.374 *p* < 0.001	*F* = 11.876 *p* < 0.001	*F* = 1.052 *p*‘= n.s.
Boys	86.64 (16.66)	89.71 (17.42)	89.33 (18.57)	84.83 (17.14)	77.89 (14.97)	75.28 (19.68)	67.33 (21.54)	73.97 (26.81)			
Total	85.49 (15.25)	89.21 (17.01)	87.55 (17.64)	82.59 (16.54)	77.28 (14.44)	73.67 (18.08)	67.90 (21.00)	71.34 (24.98)			
**Hopping for height**											
**(HH)**											
Girls	81.71 (15.74)	91.70 (19.48)	90.00 (18.85)	89.59 (21.09)	89.21 (22.42)	93.34 (19.64)	75.94 (22.90)	77.26 (25.43)	*F* = 16.729 *p* < 0.001	*F* = 66.92 *p* < 0.001	*F* = 7.048 *p* < 0.001
Boys	102.02 (12.36)	102.11 (16.29)	90.47 (20.08)	94.40 (20.71)	92.29 (20.51)	95.38 (21.14)	86.20 (19.72)	84.99 (24.20)			
Total	92.15 (17.37)	96.65 (18.74)	90.23 (19.45)	92.20 (20.99)	90.81 (21.48)	94.48 (20.47)	81.54 (21.79)	81.85 (24.91)			

**Figure 1:**
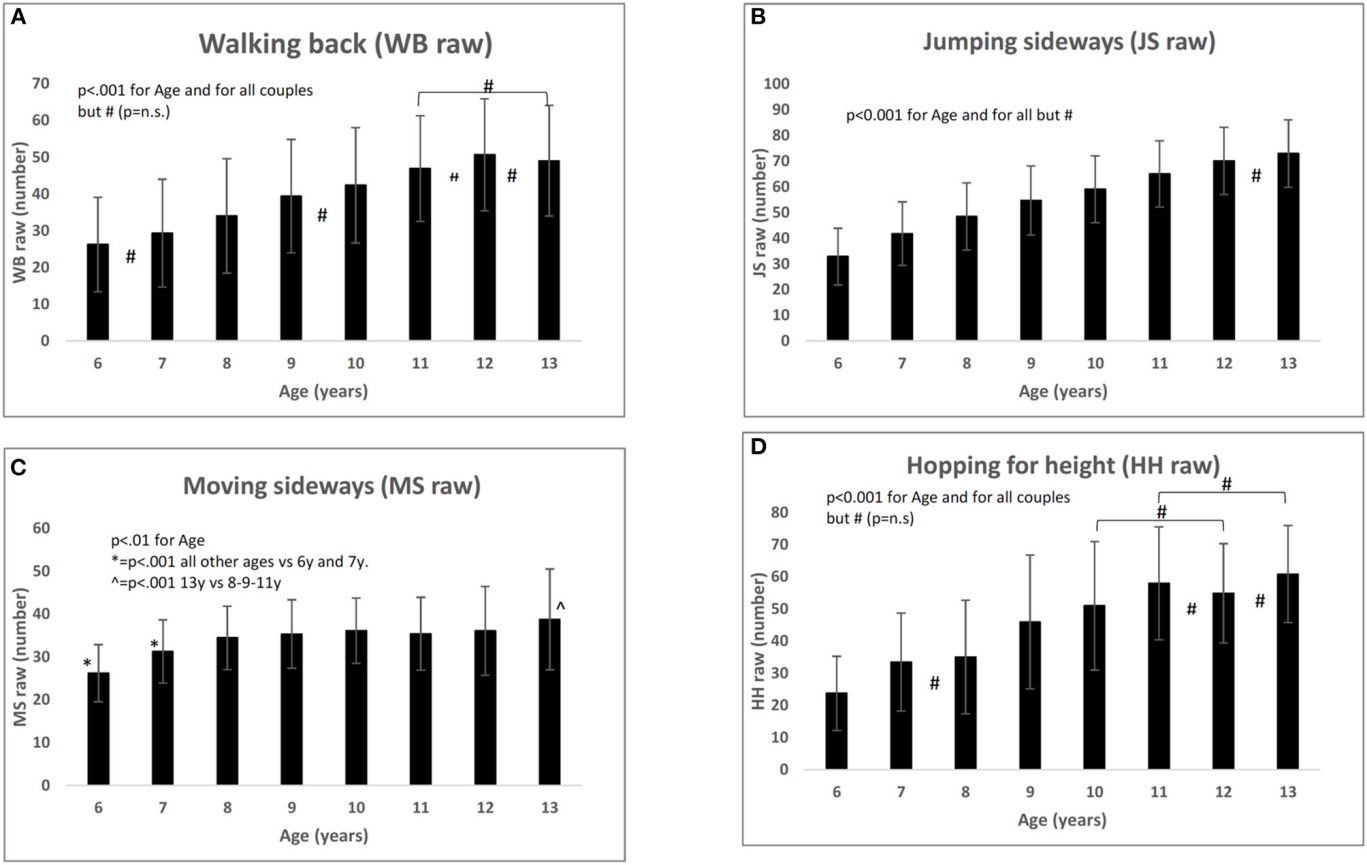
Raw values of the four subsets between 6 and 13 years. **(A)** Walking Back (WB raw); **(B)** Jumping Sideways; **(C)** Moving Sideways; **(D)** Hopping for Height.

In WB raw, girls perform better than boys (*F* = 14.52; *p* < 0.001). Globally, WB raw increased with age (*r* = 0.459; *p* < 0.001). In the *post-hoc* analysis by age group, significant differences for all but five age groups were found: 6 vs. 7 years, 9 vs. 10 years, 11 vs. 12 years, and 13, 12 vs. 13 years (Figure 1A). The standardized mean value of WB is lower than the reference (respectively, 88.33 ± 17.27 vs. 100 ± 15; *F* = 394.889; *p* < 0.001) and significantly correlates with age (Table 2), although with a value very close to zero (*r* = 0.078; *p* < 0.001).

In the other three subtests, boys performed better than girls (JS: *F* = 24.529; *p* < 0.001—MS: *F* = 9.052; *p* < 0.01—HH: *F* = 11.105; *p* < 0.001).

JS raw increased steadily with age (*r* = 0.674; *p* < 0.001), and significant differences between all age pairs were found except 12 vs. 13 years (Figure 1B). The standardized mean value of JS is no different from the reference (respectively, 99.28 ± 18.27 vs. 100 ± 15; *F* = 1.387; *p* = n.s.) and not related to age (Table 2).

MS raw grow up to 8 years (6 vs. 7 years: *p* < 0.001; 7 vs. 8 years: *p* < 0.001); between 8 and 12 years, it does not show significant differences and, finally, at 13 years is greater than all the others except for 10 and 12 years (Figure 1C). The standardized mean value of MS is lower than the reference (respectively, 80.68 ± 18.90 vs. 100 ± 15; *F* = 950.028; *p* < 0.001) and decreases with age (*r* = −0.331; *p* < 0.001).

HH raw grow regularly with age (*r* = 0.545; *p* < 0.001). Significant differences were found between all age couples except for 7 vs. 8 years, 10 vs. 12 years, 11 vs. 12 years, 11 vs. 13 years, and 12 vs. 13 years (Figure 1D). The standardized mean value of HH is lower than the reference (respectively, 90.76 ± 20.89 vs. 100 ± 15; *F* = 186.665; *p* < 0.001) and decreases with age (*r* = −0.103; *p* < 0.001).

## Discussion

The main goal of our cross-sectional research was to verify, for the first time, the current values of GMC of Italian boys and girls between 6 and 13 years of age, based on a large cohort of Italian children living in the north, center, and south of Italy. It was assumed that the current coordination levels of Italian boys and girls are lower than the reference values of the KTK battery. Secondary goals were to study gender differences, and the four subtests trend with ages. In all four subtests, we assume an increase in raw values that slowed down with increasing age. We expected that girls exhibit similar or higher values than boys in the WB. In the JS and HH, boys should show overall values higher than girls due to higher strength levels. There are no known reasons to assume gender difference in MS. Our cross-sectional search has applied the KTK test battery to a large population between 6 and 13 years. Globally, GMC values of Italian youth were lower than reference (46). Boys showed better performance than girls in MQ, RS as well as in three on four subtests.

The relevant number of studies that adopted KTK permitted us to compare our overall results (RS and MQ) not only with 1974 references (46) but also with similar researches conducted by Vandorpe et al. (45) and Moreira et al. (58). To interpret MQ values, we were able to consider the Vandorpe et al. (45) and Kiphard and Schilling (46, 47) results but not those of Moreira that did not calculate this parameter (Tables 5, 6). To compare RS and the four subtests among the four searches (Table 7), subjects between 6 and 10 years old, common to the four studies, were considered (45–47, 58). Despite this limitation, mandatory because Brazilian data were collected only for this age group, it was thought appropriate to consider Moreira data, gathered in a very different sociocultural reality but almost simultaneously with the Italian data. We compared, therefore, our data both on a worldwide geographical scale and on a time scale of about 45 years. The Kiphard 1974 German survey collected the original MQ data and was carried out on a population aged 5 to 14 years old. The Vandorpe Belgian survey investigated, in 2011, a population aged 6 to 11 years (45) while Moreira published her data from Brazilian children from 6 to 10 years old, in 2019.

**Table 5:** The number of subjects, means, standard deviations, and differences (percentages) of the motor quotient (MQ) in three comparable searches: Kiphard (46), Germany; Vandorpe et al. (45), Belgium; and our research, Italy, 2021.

**Motor quotient (MQ)**	**Number of subjects**	**Mean**	**St. dev**.	**Δ% vs. Germany**
Germany 1974	1,228	100^*^^∧^	15	—
Belgium 2011	2,470	96.5^∧^	14.3	−3.5
Italy 2021	2,206	86.64	16.22	−13.36

* *p < 0.001 vs. Belgium*,

∧ *p < 0.001 vs. Italy*.

**Table 6:** Distribution of subjects in the five levels of motor quotient (MQ level) of three comparable searches.

**Distribution in “MQ levels”**	**Italy 2020 (number)**	**Italy 2020 (%)**	**Belgium 2008 (%)**	**Germany 1974 (%)**
Bad	368	16.7	4.3	2
Low	649	29.4	16.8	14
Normal	1,110	50.3	70.2	68
Good	72	3.3	8.3	14
Excellent	7	0.3	0.4	2
Chi-square p <0.001.				

*MQ level based on MQ values: bad (56–70 MQ); low (71–85 MQ); normal (86–115 MQ); good (116–130 MQ); excellent (131–145 MQ)*.

**Table 7:** Values of RS, WB raw, JS raw, Ms raw, and HH raw, in subjects from 6 to 10 years old.

	**Mean**	**Number**	**Standard deviation**	**Between groups**	**Percentage difference vs. Germany 1974**
**Walking back**
**(WB raw)**
Germany 1974	49,477^*^^∧^^°^	677	8,121	*F* = 733.35 *p* < 0.001	—
Belgium 2011	37,632^∧^^°^	2,115	7,181		−23.94%
Brazil 2019	34,086	566	6,008		−31.11%
Italy 2021	35,059^#^	1,631	7,020		−29.14
**Jumping sideways**
**(JS raw)**
Germany 1974	52,408^∧^^°^	677	10,734	*F* = 268.513 *p* < 0.001	—
Belgium 2011	52,147^∧^^°^	2,115	9,999		n.s.
Brazil 2019	40,011	566	7,719		−23.65
Italy 2021	47,515^∧^	1,631	9,846		−9.34
**Moving sideways**
**(MS raw)**
Germany 1974	41,911^*^^∧^^°^	677	5,655	*F* = 527.406 *p* < 0.001	—
Belgium 2011	36,848^∧^^°^	2,115	4,859		−12.08
Brazil 2019	35,569°	566	5,519		−15.13
Italy 2021	33,183	1,631	4,368		−20.83
**Hopping for height**
**(HH raw)**
Germany 1974	49,479^∧^^°^	677	11,542	*F* = 344.367 *p* < 0.001	—
Belgium 2011	48,651^∧^^°^	2,115	9,891		n.s.
Brazil 2019	42,383°	566	7,535		−14.34
Italy 2021	39,014	1,631	10,306		−21.15
	**Sum of** **means**	**Number**	**Standard deviation**	**Between groups**	**Percentage difference vs. Germany 1974**
**Raw score**
**(RS)**
Germany 1974	193,275^*^^∧^^°^	677	10,070	*F* = 3,332,086 *p* < 0.001	—
Belgium 2011	175,278^∧^^°^	2,115	10,365		−9.31%
Brazil 2019	152,048	566	7,238		−21.33%
Italy 2021	154,771^∧^	1,631	9,911		−19.92%

* *p < 0.001 vs. Belgium*,

∧ *p < 0.001 vs. Brazil, °p <0.001 vs. Italy*,

# *p < 0.01 vs. Brazil*.

*Data elaborated from Kiphard (46) (Germany); Vandorpe et al. (45) (Belgium); Moreira et al. (58) (Brazil) and from our present research (Italy)*.

Regarding MQ, significant differences between the three studies (*F* = 385.832; *p* < 0.001) were found. Both Belgian and Italian results were lower than the German references (Belgium = −3.5%; Italy = −13.36%; *p* < 0.001). The Italian values were significantly lower than Belgium ones (−10.22%; *p* < 0.001). This result seems to indicate a constant GMC reduction over time. Table 6 describes, for the Italian search, the numbers and percentages of participants included in the MQ five levels. For the Belgian and German searches, the table reports only the participants' percentages at every level. The chi-square shows a significant difference in the distributions of subjects [X^2^ (8, *N* = 5,904) =654.050, *p* < 0.001] with Italian “Bad” and “Low” groups much more numerous than others.

Table 7 presents the raw values of the four subtests and RS, obtained from the four studies, with significant differences among groups (*F* = 3,332.086; *p* < 0.001). The German's RS values are higher than Belgian ones (Germany 193.28 ± 10.07; Belgium = 175.28 ± 10.37; *p* ≤ 0.001) while Italian data, lower than the first two (*p* < 0.001), are slightly higher than the Brazilian ones (Italy 154.77 ± 10.07; Brazil = 152.05 ± 7.24; *p* ≤ 0.001). Comparison among these four searches seems to confirm a downward trend in GMC and its characterization by geographical and sociocultural areas (14, 63). RS shows an increase with age that, however, tends to decrease from 11 years onward. Similar trends were shown in the original German KTK data, whereas Vandorpe et al. (45) measured a substantially linear increase. Vaccari et al. (64) identified, in young Italians of the same age, a trend with a gradual slowing increase in performance with age also in measures of balance, cardiorespiratory fitness, and lower extremity power.

A secondary goal was to study the four subtest trends in the different ages and the two genders. Our study showed Italian results below the German and Belgian values for all raw values of the four subtests (*p* < 0.001). In comparison with the Brazilian data, the Italian WB and JS subtests were better (WB: Italy = 35.06 ± 7.02; Brazil = 34.09 ± 6.01; *p* < 0.01; JS: Italy = 47.51 ± 9.85; Brazil = 40.01 ± 7.72; *p* < 0.001), while it was the opposite for MS and HH (MS: Italy = 33.18 ±4 .37; Brazil = 35.57 ± 5.52; *p* < 0.001; HH: Italy = 39.01 ± 10.31; Brazil = 42.38 ± 7.53; *p* < 0.001). Overall, the Italian standardized mean values of three out of four subtests were well below the German references (WB = −11.68%; MS = −19.32%; HH = −9.24%; *p* < 0.001). Only for JS were the Italian values similar (JS = 99.28; *p* = n.s.).

Our results showed better performance in boys than girls in the raw scores of three of the four subtests (JS: *F* = 24.529; MS: *F* = 9.052; HH: *F* = 11.105), while girls had better performances than boys in the WB (*F* = 14.52).

Overall, the four subtests showed results that do not match those that emerged from the three other searches that we adopted as a comparison. The only univocal result in all searches regarded HH, which showed higher levels of boys than those of girls. In WB, the best results of the girls were detected by our research and by Vandorpe; the German and Brazilian data, instead, showed no differences. In JS, the Italian data documented, like the German ones, better values of girls than of boys, while the Belgians and Brazilians have not found any differences. In MS, our data were the only ones to show a prevalence of the boys' performances.

For the HH test, it seems reasonable to attribute to the greater power developed by boys the cause of their better results in all searches (45, 65). The heterogeneity of the results of the other three subtests (MB, JS, and MS) suggests that the main factors potentially correlated with coordinative performance, such as the amount of PA, different types of sports practices, and body composition, but also less investigated factors such as sedentariness, sociocultural vulnerability conditions, and natural and urban environment, which act differently for girls and boys. We can, in this regard, consider a limitation of our research not having considered the many correlates and determinants of GMC. We consider, instead, a strong point of our work, the size of the population investigated in a nation, Italy, which did not have such extensive data in this field. Another element that seems relevant is the confrontation of our results with those of other searches that have used the KTK, carried out in different geographical areas and in a time ranging from 1974 to the present.

The overall picture of the results we have presented, first of all, makes us consider the continuing decline in GMC as worrying: the negative effects on health and lifestyles could weigh heavily on current generations. The significant differences between genders make us believe it appropriate to maintain a differentiated normalization. As was expected, RS increased with age, and it seems reasonable, therefore, to maintain an age-based normalization of the GMC. On the contrary, MQ tended to decrease, confirming the observations of Giuriato et al. (55). It seems that today's young people accumulate less significant motor experiences over the years, from both a quantitative (66) and qualitative (1) point of view, compared to those achieved by their peers in the 1970s. This vicious circle favors an increase in weight and fat mass that is not proportionate to the increase in height (67). However, the possibility of a reverse dynamic must also be considered, with overweight acting as a trigger to reduce PA (11).

It seems reasonable to argue, therefore, that greater use of GMC measures is useful to favor their better solicitation both in physical education and in youth sports training. It is believed that an improvement in GMC's parameters is achievable by improving both the structured physical activities and the spontaneous lifestyle of children and adolescents.

This implies a greater awareness of the reduction of this physical capacity by teachers, coaches, and policymakers. The first two will thus be able to adopt proposals for more appropriate physical activities while the latter will have a more updated and complete picture of the development needs of young people.

## Perspective

The availability of updated assessments of GMC will help physical education in schools, youth sports training, and the orientation of PA promotion policies. We believe that the KTK standards proposed by Kiphard and Schilling in 1974 (46) no longer correctly represent the benchmarks for GMC. It remains, in any case, the need to provide targets for improvement of today's results which show a wide reduction. We think, therefore, that the integration of recent results with data from the original search and the more recent past could provide new standards based on a wider population, achieving both the representativeness of different situations and the stimulus for the improvement of current coordination skills. Also, in light of the reduction of the results currently obtained by subjects up to 14 years, it seems reasonable to verify the applicability of the KTK test battery in subjects older than 14 years. If KTK were applicable in youth over 14 years, it could provide useful references to sustain policies that promote PA, sport, and physical education throughout the youth. Finally, we believe that further longitudinal research should verify the actual development of GMC with age.

## Data Availability Statement

The raw data supporting the conclusions of this article will be made available by the authors, without undue reservation.

## Ethics Statement

The studies involving human participants were reviewed and approved by Ethical Boards of the Universities of Verona (N. 2019-UNVRCLE-0298910) and Palermo (N. 8/2019) as well as the Institutional Review Board of the University of Rome Foro Italico. Written informed consent to participate in this study was provided by the participants' legal guardian/next of kin.

## Author Contributions

ML, MB, MCG, MG, VB, and FS conceptualization and methodology. MG, VB, MCG, and GB data collection. ML and MG data analysis. ML, MG, FS, MB, LG, and CB data interpretation. ML, MG, and VB writing—original draft preparation. MG, VB, and GB writing—review and editing. LG, AP, CB, and FS supervision. All authors have read and approved the final version of the manuscript and agree with the order of presentation of the authors.

## Conflict of Interest

The authors declare that the research was conducted in the absence of any commercial or financial relationships that could be construed as a potential conflict of interest.

## Publisher's Note

All claims expressed in this article are solely those of the authors and do not necessarily represent those of their affiliated organizations, or those of the publisher, the editors and the reviewers. Any product that may be evaluated in this article, or claim that may be made by its manufacturer, is not guaranteed or endorsed by the publisher.
